# ﻿A new, disjunct species of *Bahiana* (Euphorbiaceae, Acalyphoideae): Phytogeographic connections between the seasonally dry tropical forests of Peru and Brazil, and a review of spinescence in the family

**DOI:** 10.3897/phytokeys.219.95872

**Published:** 2023-02-20

**Authors:** Kenneth J. Wurdack

**Affiliations:** 1 Department of Botany, MRC-166, National Museum of Natural History, Smithsonian Institution, P.O. Box 37012, Washington DC 20013-7012, USA National Museum of Natural History Washington DC United States of America

**Keywords:** Biogeography, Huancabamba Depression, molecular phylogeny, SDTF, spines, taxonomy

## Abstract

*Bahiana* is expanded from 1 to 2 species with the description of *B.occidentalis* K. Wurdack, **sp. nov.** as a new endemic of the seasonally dry tropical forests (SDTFs) of Peru. The disjunct distribution of *Bahiana* with populations of *B.occidentalis* on opposite sides of the Andes in northwestern Peru (Tumbes, San Martín) and *B.pyriformis* in eastern Brazil (Bahia) adds to the phytogeographic links among the widely scattered New World SDTFs. Although *B.occidentalis* remains imperfectly known due to the lack of flowering collections, molecular phylogenetic results from four loci (plastid *matK*, *rbcL*, and *trnL-F*; and nuclear ITS) unite the two species as does gross vegetative morphology, notably their spinose stipules, and androecial structure. Spinescence in Euphorbiaceae was surveyed and found on vegetative organs in 25 genera, which mostly have modified sharp branch tips. Among New World taxa, spines that originate from stipule modifications only occur in *Bahiana* and *Acidocroton*, while the intrastipular spines of *Philyra* are of uncertain homologies.

## ﻿Introduction

Seasonally dry tropical forest (SDTF) is a biome broadly characterized by a pronounced dry season in addition to low mean annual precipitation, flora with diverse drought adaptations (e.g., deciduousness, succulence), fertile non-acidic soils, fire intolerance, and sparse herb layers with few grasses (Pennington et al. 2000, 2009; Banda-R et al. 2016). SDTFs have attracted much recent attention and research due to their rich endemism, interesting historical biogeography, and conservation concern (Pennington et al. 2009). In the New World they range from northwestern Mexico, through the Caribbean, and across South America, roughly encircling the Amazon basin. Within this range they are patchy, especially along the Pacific side and inter-Andean valleys of South America, and are broken into floristic “nuclei” which display patterns of regional endemism and a strong geographic component to floristic relatedness. Taxa that are widespread or disjunct across their breadth are rare, but some distribution patterns and modeling suggest SDTF formations were more continuous across South America during the Pleistocene (Pleistocene Arc Theory; see Werneck et al. 2011). The processes of SDTF flora assembly and species diversification are thought to revolve around relatively long biome stability, dispersal limitations, and phylogenetic niche conservatism (Pennington et al. 2009).

Euphorbiaceae are an important component of SDTF floras and in terms of diversity are among the top six most species-rich families in woody plant inventories (Banda-R et al. 2016). The family is represented by diverse endemics including *Bahiana* spp., *Croton* spp., *Gymnanthesboticario* Esser, M.F.Lucena & M.Alves, and a small species radiation of stem-succulent *Euphorbia* spp. in eastern Brazil (Hurbath et al. 2021). *Bahiana* J.F.Carrión was recently described as a monotypic genus and narrow SDTF endemic, with a population of ca. 20 plants at the type locality in central Bahia of eastern Brazil (Carrión et al. 2022). A cryptic plant from the Peruvian SDTF, here described as a new species of *Bahiana*, that was first collected 40 years ago (1982) and is now represented by 35 collections, has lain unidentified to genus (or misidentified) among Euphorbiaceae in the Missouri Botanical Garden herbarium. The principal impediment to recognition of this taxon has been that many of those collections which were made for plot studies are sterile, and the reproductive collections are either fruiting or else in very young staminate buds. I recognized five years ago that these collections were united by leaf architecture and spinose stipules, and represented an unusual, potentially undescribed Euphorbiaceae-Acalyphoideae. However, an initial analysis of plastid *trnL-F* sequence data within the context of the family-wide phylogeny of Wurdack et al. (2005) only yielded placement with New World members of Acalyphoideae tribe Bernardiaeae (clade A7; Bernardia clade, Carrión et al. 2022; Bernardieae pro parte, Radcliffe-Smith 2001; Webster 2014), with no clear generic affiliation. The phylogenetic analysis and description of *Bahianapyriformis* by Carrión et al. (2022) expanded the sampling for the Bernardia clade and provided critical context for the Peruvian plant. The four genera comprising the Bernardia clade are diverse, even in habitat, where species of *Adenophaedra* (Müll.Arg.) Müll.Arg. and *Caryodendron* H.Karst. are in rainforests, *Bahiana* are in SDTF, and *Bernardia* Houst. ex Mill. are wide ranging from rainforests to dry-adapted taxa, including SDTF endemics.

Although the Peruvian plants remain incompletely known, morphological similarities and new molecular phylogenetic results indicate that they should be recognized as a second species of *Bahiana*. Hopefully its description will spur further efforts to secure flowering collections and additional localities. Moreover, it adds an unusual floristic connection among the SDTFs of South America. Given the fragmented and sometimes erroneous information on spinescence in Euphorbiaceae and its relevance to ecology (i.e., understanding the evolution anti-herbivory defenses), the character was reviewed for the entire family, with special emphasis on spinose stipules to enable comparisons with *Bahiana*.

## ﻿Materials and methods

Molecular methods for DNA extraction with modified Qiagen DNeasy Plant kits, then amplification and fluorescent Sanger sequencing with BigDye Terminator v3.1 chemistry (Thermo Fisher Scientific, Waltham, Massachusetts) on an ABI 3730xl DNA Analyzer (Thermo Fisher Scientific) followed prior studies (i.e., Wurdack et al. 2005; Wurdack and Davis 2009; Cardinal-McTeague et al. 2019; van Welzen et al. 2021). The 2-marker (plastid *rbcL*, *trnL-F*) data set of Carrión et al. (2022) was largely derived from Wurdack et al. (2005) by restricting the taxon sampling to Acalyphoideae and adding *Bernardia* clade representatives, and then further modified here by my addition of three tips (two of the Peruvian *Bahiana* and previously published *Adeliacinerea* [Wiggins & Rollins] A.Cerv., V.W.Steinm. & Flores Olv., GenBank DQ997801, HG971805). *Adelia* may not be monophyletic (De-Nova and Sosa 2007; Cervantes et al. 2016), and the addition of *A.cinerea* to the 2-marker data set provides better context for the use of that outlier species as an outgroup in the 4-marker data set. The 4-marker data set of Carrión et al. (2022) included *Bernardia* clade representatives for plastid *matK* (including 3’ *matK-trnK*), *petD*, and *trnL-F*, and nuclear ribosomal ITS, and was modified here by my addition of the Peruvian *Bahiana* (two tips). Laboratory work on each of the two Peruvian samples occurred at very different times and under contamination-control protocols developed for degraded museum samples. The visually best-preserved (“greenest”) of the 35 collections (*A. Gentry et al. 37824*, MO) yielded DNA of sufficient quality to sequence *matK* (partial), *rbcL*, *trnL-F*, ITS, and ETS (GenBank OP900956, OP900955, OP900957, OP879622, OP901195, respectively); more degraded DNA from another collection (*C. Díaz S. et al. 6545*, US) yielded sequences for *trnL-F*, ITS, and ETS (GenBank OP900958, OP879623, OP901196). Due to the lack of differences in ITS between the new species accessions, a 400 bp fragment of ETS was sequenced to further explore population variation. Nuclear ribosomal ETS has been a useful rapid-evolving complement to ITS for fine-scale resolution of many clades of Euphorbiaceae (e.g., Cardinal-McTeague et al. 2019; van Welzen et al. 2021). For new data, I amplified and/or sequenced the genetic markers using mostly standard primers for *matK* (400f, 1159r, 1053f, K2r), *rbcL* (1f, 724r, 626f, 1360r), *trnL-F* (c, d, e, f, intF), ITS (5a, P3, U2, U4), and ETS (F2; 18s_5pr, newly published here, CTGGCAGGATCAACCAGGTAGCA).

The reads were assembled with Sequencher v5.2.4 (Gene Codes, Ann Arbor, Michigan, U.S.A.) and consensus sequences were manually inserted into the multiple sequence alignments (MSAs) of Carrión et al. (2022), followed by alignment refinements based on a sequence similarity criterion. Preliminary analyses showed that my minor MSA improvements and various stringencies in the masking of ambiguously aligned regions had limited impact on resolution or support values, thus a limited exclusion set of select indel hotspots was implemented (details in the archived MSAs). The 2-marker data set had 418 columns excluded (original length 3230 bp) and 24.7% missing data; the 4-marker data set had 260 columns excluded (original length 6560 bp) and 22.2% missing data. Maximum likelihood (ML) analyses used IQ-TREE v1.6.11 under GTR+F+I+G4 and clade support estimated by 1000 rapid bootstrap replicates (Trifinopoulos et al. 2016). Bayesian inference (BI) was with MrBayes v3.2.7a (Ronquist et al. 2012) as implemented on CIPRES XSEDE with two concurrent runs, each with four chains and sampling every 1000 generations over 50 000 000 generations, a 0.2 temperature coefficient, a conservative 25% burn‐in, and an Effective Sample Size (**ESS**) > 200 verified with Tracer v1.6.0 (Rambaut et al. 2013).

Scanning electron microscopy (**SEM**) used a Zeiss EVO MA15 (Carl Zeiss SMT, Inc., Peabody, Massachusetts) at 3 kV after sputter-coating herbarium specimen fragments with Au/Pd over C (11 nm total) using a Leica EM ACE600 (Leica Microsystems GmbH, Wetzlar, Germany). Staminate inflorescences were rehydrated and buds microdissected before critical point drying (**CPD**) from an ethanol transition. A leaf was cleared in 5% sodium hydroxide followed by saturated chloral hydrate, and then stained with basic fuchsin (1% in absolute ethanol). Light microscopy (**LM**) was with a Leica DM6 B (Leica Microsystems Inc., Deerfield, IL) or an Olympus DSX100 (Olympus Corp., Tokyo, Japan). Spinescence was assessed based on literature reports (mostly confirmed with collections), surveys of herbarium specimens (primarily MO, NY, US, and type images in JSTOR Global Plants, https://plants.jstor.org/), and observations of living plants. Words pertaining to spinescence were searched for in literature treatments (e.g., Webster 2014) of Euphorbiaceae as leads to additional taxa. Not considered here were trichomes, spines (or horns) associated with reproductive structures such as fruit pericarps (e.g., *Hancea* Seem., *Mallotus* Lour., *Microstachys* A.Juss., *Sclerocroton* Hochst.) and bracts (e.g., *Dalechampia* Plum.), or sub-spinose branch tips that appear to be the result of weathering (e.g., *Bernardiaobovata* [Chodat & Hassl.] Pax & K.Hoffm.) rather than developmental processes. Many pericarp spinose structures appear more ornamental in nature in being few (1–2 per valve), short, and/or blunt. Spinose species estimates are uncertain in *Euphorbia* L., and there is some subjectivity in the distinctions between spinose and sub-spinose structures (e.g., variations in *Erythrococca* spp. stipules).

### ﻿Phylogenetic results

**Figure 1.**
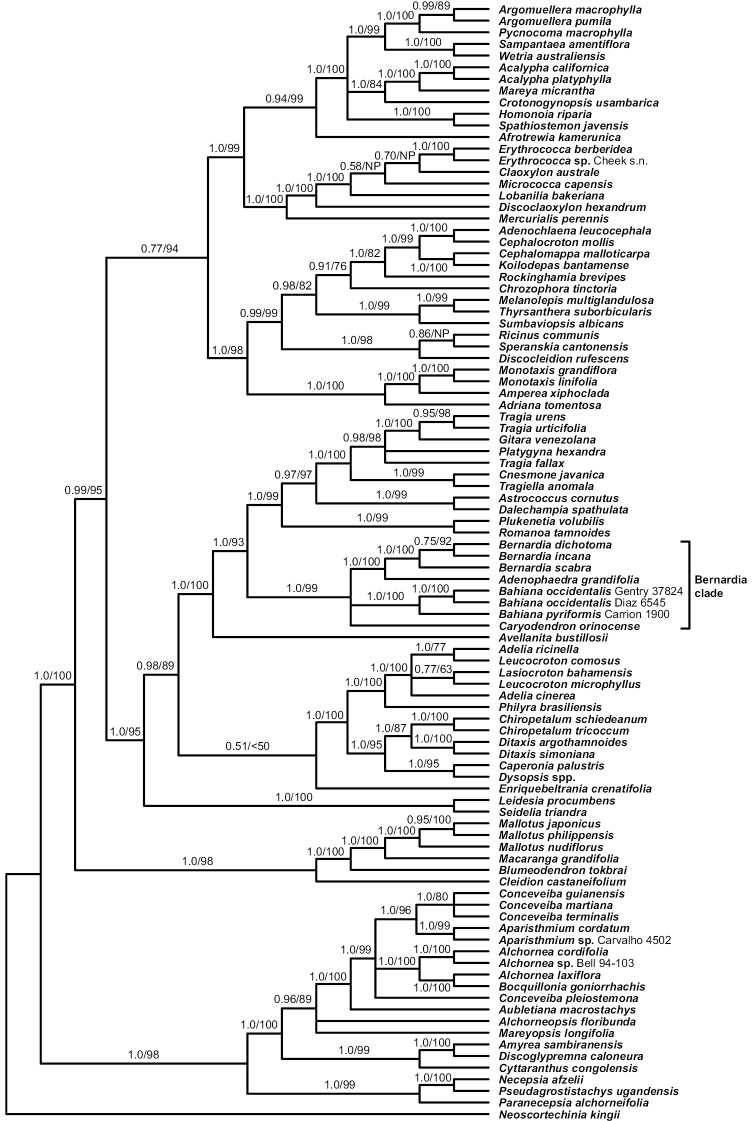
Phylogenetic relationships of *Bahiana* and its Acalyphoideae relatives. Bayesian 50% majority-rule consensus tree based on the combined 2-marker (*rbcL*, *trnL-F*), 96-tip data set with posterior probability/ML bootstrap values indicated, respectively. NP = an edge not present with ML.

**Figure 2.**
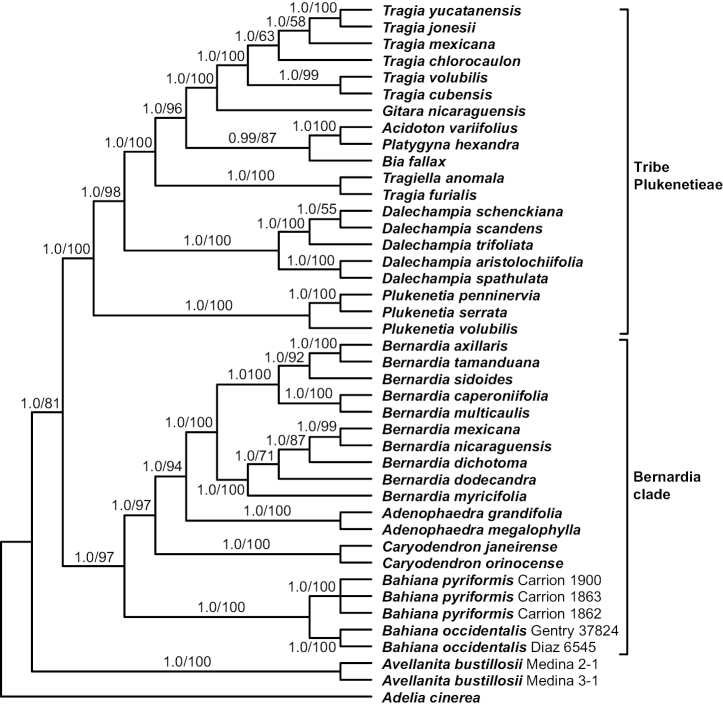
Phylogenetic relationships of *Bahiana* and its Bernardia clade relatives. Bayesian 50% majority-rule consensus tree based on the combined 4-marker (*matK*, *petD*, *trnL-F*, ITS), 42-tip data set with posterior probability/ML bootstrap values indicated, respectively.

The two geographically widely separated samples of the Peruvian *Bahiana* have identical ITS and *trnL-F* sequences, but differ at four positions in ETS (1.0% difference); slower evolving *matK* and *rbcL* were not compared because sequences were not generated for both samples. These two samples also provide molecular evidence to unite staminate and pistillate collections. Within the context of the modified Carrión et al. data sets, the new species is strongly supported (posterior probabilities, PP = 1.0; bootstrap percentages, BP = 100%) as sister to *Bahianapyriformis* within a similarly supported *Bernardia* clade, and branch lengths (not shown) indicate considerable sequence divergence between the two species (Figs 1, 2).

## ﻿Data resources

The data underpinning the phylogenetic analyses reported in this paper are deposited in GenBank and the Dryad Data Repository at https://doi.org/10.5061/dryad.wstqjq2r6.

## ﻿Taxonomy

### ﻿ Bahiana occidentalis

Taxon classificationPlantaeMalpighialesEuphorbiaceae

K. Wurdack sp. nov.

59315A1C-8E75-5E6F-9618-5B5E8995C9DA

urn:lsid:ipni.org:names:77314428-1

Figs 3 , 5

#### Diagnosis.

Differs from *Bahianapyriformis* in leaves smaller (3.5–6[8.1] × 2.1–3.9[4.6] versus 6–12 × 3–6 cm), staminate cymules 1-flowered (versus usually 3-flowered), fruits smaller and subglobose (ca. 9 × 14 mm versus 18–28 × 17–23 mm and usually obovoid to pyriform), fruit pedicels longer (10–18 versus up to 5 mm), and seeds smaller (6.9–7.3 long × 6.4–6.5 wide × 6.8–7.4 thick versus 10–15 long × 8–11 wide × 8–13 thick mm).

#### Type.

Peru. Tumbes: Zarumilla Province, Matapalo, zona “El Caucho-Campo Verde”, Parcela 2 × 500 m (evaluación florística) paralela a parcela “V” de evaluación forestal permanente, desde 420 m hasta 500 m, 03°50'29"S, 080°15'30"W (−3.8413800, −80.2583300), 500 m, 11 Feb 1993 (fr), *C. Díaz S. et al. 6288* (holotype: MO sheet 7004543; isotypes: K, NY, US, USM; 13 reported duplicates).

#### Description.

Small trees, 4–10 m, trunk to 12 cm dbh, probably dioecious (collections unisexual); bark of branches smooth, lenticellate; lateral leafy branchlets 1–6 cm long, 1–2 mm wide, terete, sometimes as brachyblasts with numerous compressed nodes, or with zones of compressed nodes (long shoot/short shoot transitions on same axis), sparsely pubescent when young. ***Indumentum*** simple, pale, to 0.5 mm long. ***Stipules*** free, persistent, paired; when young appressed to stem, triangular 1.5–2.5 × 1 mm, with prominent midrib and narrow membranous margins extending ca. 0.1 mm, sparsely short puberulent; with age spinose, stiff, accrescent to 5–6 mm long, base with shield-like attachment zone to stem, projecting 45–90° from stem, glabrescent; resting buds with multiple series of spines and scales; bud scales 1.5 × 1 mm, triangular, navicular. ***Leaves*** alternate, simple, petiolate. ***Petioles*** 3–7(11) mm long, 0.5–0.7 mm tall × 0.7–1 mm wide (mid-length cross section), slightly dorsiventrally flattened (rarely terete), adaxially moderately pubescent and abaxially distinctly less pubescent to nearly glabrous. ***Leaf blades*** elliptic, 3.5–6(8.1) × 2.1–3.9(4.6) cm, length:width ratio 1.45–2.91:1 (mean = 2.00, SD = 0.313, n = 55, 5 leaves each from 11 collections); base obtuse to acute; apex obtuse to acute, tip minutely retuse and tipped by a globose gland as on marginal teeth; chartaceous, margins subentire proximally (near base obscurely crenate with little evidence of teeth) to distinctly toothed (crenate) distally, tooth depth varying 0.2–0.5 mm, 7–15 well-defined teeth per side, tooth tip bearing persistent sub-globose glandular knob to 0.2 mm diam. that terminates principal (tertiary) vein; laminar glands (cicatricose-crateriform glands sensu Cervantes et al. 2009) abaxial, scattered, 5–20 glands per leaf, elliptic, 0.1–0.2 × 0.1–0.25 mm, associated with tertiary or quaternary veins; other laminar or petiolar glands absent; trichomes abaxially usually only associated with major veins (rarely uniformly pubescent, *A. Gentry et al. 37824*), sparsely present along midvein but more densely as acarodomatial tufts in axils of primary/secondary vein junctions; acarodomatia with narrow flaps of vein tissues extending to 0.1 mm from vein junctions, not coincident with laminar glands; adaxial surface micropustulate (likely an artifact of drying around subsurface crystals), pustules mostly tracing vein fabric. ***Venation*** pinnate, agrophic veins absent, major secondaries bronchidodromous, 5–7 secondary veins per side, intersecondaries absent, tertiary and quaternary veins mixed percurrent. ***Staminate inflorescences*** axillary and appearing terminal, 6–11 mm long in young bud, racemose, simple, ca. 20+ cymules in 3 anticlockwise spirals, cymules each containing 1 bud, subtended by a bract and 2 lateral bractlets; bract 1.5 × 1 mm, navicular, tip acuminate; bractlets navicular, 1.7 × 0.4 mm, glabrous internally and hirsute externally. ***Staminate flowers***: sepals 5?, densely hirsute externally, glabrous internally; receptacle glandular, hirsute; stamens 12–15, filaments free; anthers dorsifixed, introrse, pollen sacs unequal with dorsal pair longer than ventral, connective with protrusion; petals and disc segments absent. ***Pistillate infructescences*** axillary, with 1–2 nodes, dichotomously branched, 1–2 fruits per inflorescence; proximal internode (peduncle) 10–40 mm long; ultimate branches (pedicels) 10–18 mm long, 0.5–0.6 mm diam., articulated and often bent at middle but not detaching there, proximal segment (relative to articulation) 3–9 mm long, distal segment 4–8 mm long, slightly thicker and usually darker colored; nodes subtended by navicular bract to 2 mm long. ***Pistillate flowers*** (details inferred from fruits) sepals 5, 1.5 × 1–1.5 mm, triangular, slightly unequal in size, interior glabrous, disk annular, thin, densely hirsute; locules 3, styles 3, undivided, thin, to 2.5 mm long, not connate into a column, glabrous. ***Fruits*** schizocarps, subglobose, trilobed, ca. 9 (long) × 14 (wide) mm, splitting septicidally and loculicidally into 3 equal 2-valved mericarps; valve segments ca. 13 × 5 mm; sepals and styles persistent; sparsely pubescent, pericarp dry; endocarp woody, 0.8–1 mm thick (equatorial at dorsal dehiscence suture); epicarp ca. 0.2 mm thick, separating when dry, inner surface vascularized, colliculate internally and externally; septa of mericarps thin, nearly complete except for distal funicular gap, shallow basal triangle 4 × 1 mm; columella 5–6 mm long, 1.2–1.3 mm wide (middle), trigonous, tip retaining arms to 1–1.5 mm long, persistent. ***Seeds*** 1 per chamber, ellipsoid, laterally slightly compressed, 6.9–7.3 (long) × 6.4–6.5 (wide) × 6.8–7.4 (thick) mm, minutely apiculate for 0.2–0.4 mm; testa ca. 0.2 mm thick, dry, smooth, obscurely brown-marbled; ecarunculate, raphe ventral; hilum near top, triangular, ca. 1.5–2 × 1.5–2 mm; embryo axile spathulate, straight, stalk 1.2 × 0.6 mm, cotyledons ovate, 4.5 (long) × 4 (wide) × 0.1 (thick) mm, with midrib and higher order veins evident, nearly filling seed profile; endosperm firm, oily; central cavity absent.

#### Etymology.

The specific epithet *occidentalis* refers to western, and indicates the distribution of the new species in western South America, in contrast to *B.pyriformis*, which grows on the eastern side of the continent.

#### Distribution and ecology.

The populations of *Bahianaoccidentalis* in Tumbes and San Martín are separated by more than 520 km and represent two (equatorial and eastern, respectively) of the three SDTF subunits defined for Peru (Linares-Palomino 2006). The new species is expected also to occur in adjacent Ecuador, which contains extensions of the Tumbesian SDTF, and perhaps also in the Marañón inter-Andean STDF that is in between the two populations, although it is isolated from them and mostly at a higher elevation. It occurs in lowland SDTF (500–720 m in Tumbes, 350 m in San Martín). Seasonal rains typically fall (Dec-) Jan to Apr (85% of annual precipitation; Cueva-Ortiz et al. 2020), after critically dry Sep to Nov. Flowering appears to coincide with the start of the rainy season (young staminate inflorescences present until mid-Nov) and mature fruit by Feb. The collections, although not diagnostic due to having only very young inflorescences or fruits, appear unisexual, which probably indicates dioecy as has been documented for *B.pyriformis* (Carrión et al. 2022). The Tumbesian SDTF appears to have few other endemic Euphorbiaceae (e.g., *Acalyphadelicata* Cardiel, *Crotontumbesinus* Riina) (Cardiel 2006; Linares-Palomino et al. 2010; Feio et al. 2018).

#### Conservation status.

Following the criteria and categories of IUCN (2012), *B.occidentalis* is given a preliminary status of Vulnerable (VU) under geographic range criteria B2 area of occupancy < 2000 km^2^ (B2a, known to exist at no more than 10 locations; B2b, continuing decline projected). The Tumbes population is within the Reserva Nacional de Tumbes, a protected area of relatively pristine forest and will ensure long term conservation of the taxon. However, the eastern SDTFs in San Martín are fragmented due to farming, including in the area around the Gentry collection locality (fide Google Earth imagery). *Bahianapyriformis* is presently known from a single, small population in a mostly deforested region that is not protected and still at risk.

**Figure 3.**
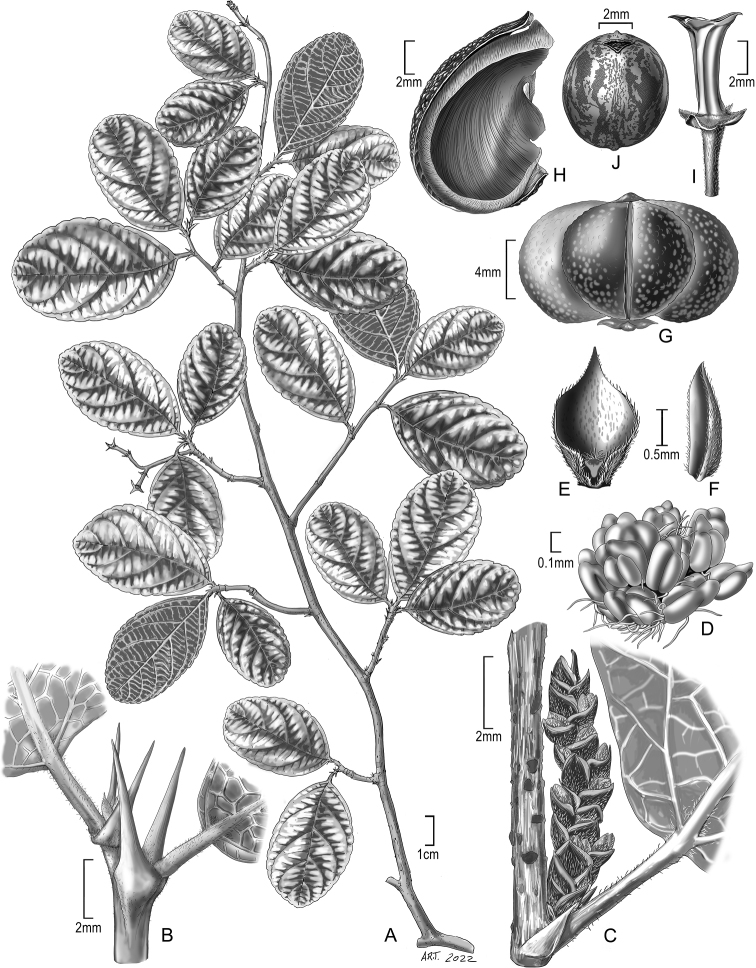
Illustration of *Bahianaoccidentalis***A** habit **B** shoot tip with spinose stipules **C** staminate inflorescence in bud **D** androecium **E** staminate bract **F** staminate bractlet **G** fruit **H** fruit valve (coccus) **I** columella **J** seed (ventral). Sources: **A, B, H–J***C. Díaz et al. 6288*, US **C–F***C. Díaz et al. 6148*, MO **G***C. Díaz et al. 7340*, MO.

**Figure 4.**
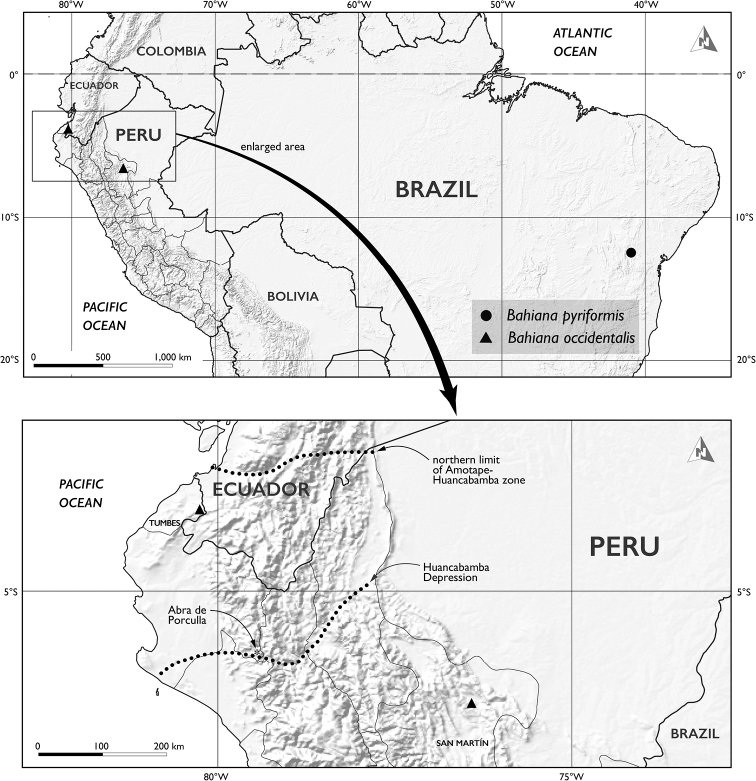
Distribution of *Bahiana* in northern South America (triangle, *B.occidentalis*; circle, *B.pyriformis*). Each marker represents multiple collections. Southern limit of the Amotape–Huancabamba zone is beyond the boundary of lower map.

**Figure 5.**
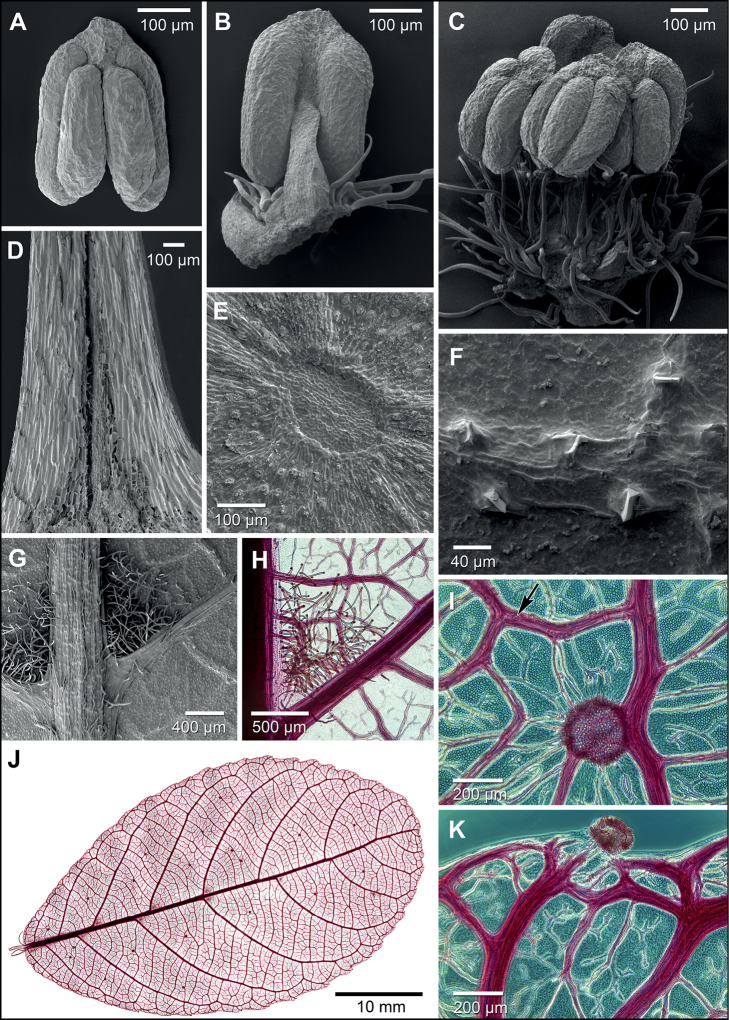
Morphology of *Bahianaoccidentalis***A** anther ventral **B** stamen dorsal **C** androecium with outer stamens removed to show hirsute receptacle (young bud) **D** stipule inner structure (split longitudinally) **E** leaf gland (abaxial) **F** leaf surface with prismatic crystals poking through epidermis along veins (adaxial) **G** leaf acarodomatia bounded by primary and secondary veins (abaxial) **H** leaf acarodomatium (clearing) **I** leaf gland with crystal along vein indicated by arrow (clearing) **J** whole leaf (clearing; tiled from 418 images) **K** glandular tooth at leaf margin (clearing) **A–G**SEM**H, J** brightfield LM**I, K** darkfield LM; sources **A–C***C. Díaz S. et al. 5522*, MO **D***C. Díaz S. et al. 6148*, MO **E–K***C. Díaz S. et al. 6288*, US.

#### Additional specimens examined.

**Peru. San Martín**: 31 km S of Tarapoto, dry forested slopes overlooking Río Huallaga, Transect 1, 06°35'S, 076°25'W (−6.5833333, −76.4166667), 350 m, 18 Jul 1982, *A.H. Gentry et al. 37732* (MO sheet 3029763); *ibid. loc.*, Transect 3, 20 Jul 1982 (stam infl), *A.H. Gentry et al. 37824* (MO sheet 3211186). **Tumbes**: Zarumilla, Matapalo. Entre P.C. “El caucho” y P.C. “Campoverde”, Bosque Nacional de Tumbes, Reserva de Biósfera del Noroeste, Arbol #326, 03°50'29"S, 080°15'33"W (−3.8413889, −80.2591667), 720 m, 21 Jul 1992, *C. Díaz S. et al. 5009* (MO sheet 5564626); *ibid. loc.*, Arbol #359, 22 Jul 1992, *C. Díaz S. et al. 5072* (MO sheet 6060058). Parcela “V” de evaluación permanente, No. 393, 03°50'29"S, 080°15'33"W (−3.8413800, −80.2591600), 720 m, 27 Oct 1992, *C. Díaz S. et al. 5176* (MO sheet 6060054); *ibid. loc.*, No. 427, 27 Oct 1992, *C. Díaz S. et al. 5201* (MO sheet 6060056); *ibid. loc.*, No. 641, 27 Oct 1992 (stam infl), *C. Díaz S. et al. 5390* (MO sheet 5707417); *ibid. loc.*, No. 658, 31 Oct 1992 (stam infl), *C. Díaz S. et al. 5432* (MO sheet 5707423); *ibid. loc.*, No. 666, 31 Oct 1992 (stam infl), *C. Díaz S. et al. 5438* (MO sheet 5707422); *ibid. loc.*, No. 679, 31 Oct 1992 (stam infl), *C. Díaz S. et al. 5472* (MO sheet 5707421); *ibid. loc.*, No. 719, 2 Nov 1992 (stam infl), *C. Díaz S. et al. 5486* (MO sheet 5707420); *ibid. loc.*, No. 720, 2 Nov 1992, *C. Díaz S. et al. 5487* (MO sheet 5707415); *ibid. loc.*, No. 732, 2 Nov 1992, *C. Díaz S. et al. 5489* (MO sheet 5707416); *ibid. loc.*, No. 735, 2 Nov 1992, *C. Díaz S. et al. 5491* (MO sheet 5707419); *ibid. loc.*, No. 721, 2 Nov 1992, *C. Díaz S. et al. 5520* (MO sheet 5707428); *ibid. loc.*, No. 725, 2 Nov 1992, *C. Díaz S. et al. 5521* (MO sheet 6060063); *ibid. loc.*, No. 729, 2 Nov 1992 (stam infl), *C. Díaz S. et al. 5522* (MO sheet 6060062); *ibid. loc.*, No. 743, 2 Nov 1992, *C. Díaz S. et al. 5537* (MO sheet 5707424); *ibid. loc.*, No. 326, 10 Nov 1992, *C. Díaz S. et al. 5957* (MO sheet 6060061); *ibid. loc.*, No. 359, 10 Nov 1992, *C. Díaz S. et al. 5979* (MO sheet 6060060). Parcela de evaluación florística (2 mt. × 500 m) paralela a la parcela “V”, orientación Este-Oeste, 03°50'29"S, 080°15'33"W (−3.8413800, −80.2591600), 500 m, 12 Nov 1992, *C. Díaz S. et al. 6007* (MO sheet 6060059); *ibid. loc.*, 12 Nov 1992, *C. Díaz S. et al. 6127* (MO sheet 6060052); *ibid. loc.*, 12 Nov 1992 (stam infl), *C. Díaz S. et al. 6148* (MO sheet 5707407); *ibid. loc.*, 12 Nov 1992, (stam infl), *C. Díaz S. et al. 6149* (MO sheet 6060065); *ibid. loc.*, 12 Nov 1992, *C. Díaz S. et al. 6151* (MO sheet 6060064). Entre P.C. “El caucho” y P.C. “Campoverde,” Bosque Nacional de Tumbes, Reserva de Biósfera del Noroeste, Arbol 326, 03°50'29"S, 080°15'33"W (−3.8413800, −80.2591600), 720 m, 21 Jul 1992, *C. Díaz S. et al. 5009* (MO sheet 5564626); Arbol #359, 22 Jul 1992, *C. Díaz S. et al. 5072* (MO sheet 6060058). Zona “El Caucho-Campo Verde”. Parcela 2 × 500 m (evaluación florística) paralela a parcela “V” de evaluación forestal permanente, 03°50'29"S, 080°15'30"W (−3.8413800, −80.2583300), 500 m, 11 Feb 1993, *C. Díaz S. et al. 6272* (MO sheet 6060051); *ibid. loc.*, 11 Feb 1993, *C. Díaz S. et al. 6277* (MO sheet 6060053); *ibid. loc.*, 11 Feb 1993, *C. Díaz S. et al. 6282* (MO sheet 6060057). Zona “El Caucho-Campo Verde”, Parcela “E” evaluación permanente, No. 326, 03°50'29"S, 080°15'30"W (−3.8413800, −80.2583300), 500 m, 16 Feb 1993 (fr), *C. Díaz S. et al. 6545* (MO, US); *ibid. loc.*, 17 Feb 1993, *C. Díaz S. et al. 6575* (MO sheet 6060055); *ibid. loc*., No. 359, 17 Feb 1993, *C. Díaz S. et al. 6605* (MO sheet 6060066); *ibid. loc.*, 17 Jan Feb 1995 (fr), *C. Díaz S. et al. 7430* (MO sheet 5707408).

## ﻿Discussion

Despite being relatively well-collected, *Bahianaoccidentalis* remains imperfectly known due to the lack of flowering specimens and limitations with inferring floral details from buds and fruits. Pistillate flowers are unknown, and the anthers are too underdeveloped for pollen comparisons. Characteristics shared by both *Bahiana* spp., which in combination are not in any other genus of Euphorbiaceae, include persistent spinose stipules, staminate bracts of two orders, 12–15 free stamens with dorsifixed apiculate anthers, and slender undivided styles (see generic comparisons in Carrión et al. 2022: table 2). Especially distinctive are the spinose stipules, a feature present in only five genera of Euphorbiaceae, of which only *Bahiana* and unrelated *Acidocroton* (Crotonoideae) have spiny species in the Neotropics (see below). Other *Bernardia* clade genera have stipules that are sheathing and caducous (*Caryodendron*); or mostly small, triangular, sometimes thickened, and persistent or tardily deciduous (*Adenophaedra*, *Bernardia*).

Beyond biogeography there are clear morphological differences in staminate cymules, fruits, and leaves that serve to distinguish the two species of *Bahiana*, and additional differences may be discovered when flowering collections become available. Dissection of young staminate inflorescences found each cymule of *B.occidentalis* contained a single bud, while *B.pyriformis* is described as “cymules usually 3-flowered” with an early-developing central flower (Carrión et al. 2022). Within the *Bernardia* clade, the fruits (and seeds) of *B.occidentalis* are closer in size to small-fruited *Adenophaedra* and most *Bernardia* (*B.macrocarpa* A.Cerv. & Flores Olv. is also large-fruited), rather than large-fruited *Bahianapyriformis* and *Caryodendron* spp. The leaves of both species of *Bahiana* are similar in details of simple indument type, abaxial leaf glands, gland-tipped teeth, and paracytic stomata (Fig. 5E–K; Carrión et al. 2022: Fig. 3). They notably differ in leaf size and some anatomical characters, with *B.pyriformis* possessing numerous relatively large crystalline druses that do not trace the vein fabric, whereas *B.occidentalis* has small epidermal prismatic crystals that follow the venation (Fig. 5I). The crystals in *B.occidentalis* often pierce the surface of the dried leaves (Fig. 5F) and cause rough, finely pustulate adaxial surfaces; similar crystals occur in *Acalypha* L. (Cardiel et al. 2020). Each tooth is capped by a sub-globose marginal gland with a palisade epidermis that resembles a colleter of the standard (S) type, but is not elongate or stalked (Fig. 5K) as seen in other Euphorbiaceae (Thomas 1991; Vitarelli et al. 2015). There is little morphological distinction between the Tumbes and San Martín collections of *B.occidentalis*, except for more pronounced brachyblasts in the latter. While one San Martín collection (*A. Gentry 37824*) is noteworthy in abaxially uniformly pubescent leaves, a second collection (*A. Gentry 37732*) from the same locality is typical of the species with sparse pubescence (except the defined hirsute acarodomatia). Associated with the acarodomatia along the primary vein are hairs, but almost no pocket (merely a narrow flange from the bounding veins), and no pits or glands (Fig. 5G, H).

The biogeography of *Bahiana* is notable due to its substantial disjunction (Fig. 4). The two species are nearly 4000 km apart at their closest locations in Peru and Brazil, and the populations of *B.occidentalis* in Tumbes and San Martín are separated by more than 520 km. The known distribution of *B.occidentalis* is within the floristically unusual Amotape–Huancabamba zone (see Weigend 2002), and the two populations are on opposite sides of the Andes across the Huancabamba Depression which is in the center of the zone. The Huancabamba Depression, the lowest point in the Andean chain (2145 m at Abra de Porculla), is a dispersal impediment to montane species but has been considered an opportunity for lowland SDTF taxa (e.g., *Bahiana*) to cross the Andes (Linares-Palomino et al. 2003; Quintana et al. 2017). Estimates of diversification histories for taxa in this region have been varied. Pennington et al. (2010) found the SDTF endemic *Cyathostegiamatthewsii* (Benth.) Schery (Fabaceae) had relatively high sequence divergences among inter-Andean populations, which indicated an older diversification history and in particular, populations (Loja and Marañón) spanning the Huancabamba Depression were isolated 2.8 (+/- 0.6) million years. In *B.occidentalis*, the low genetic divergences (0–1% across three fast-evolving loci) between the two populations suggest recent dispersal; however, broader genomic comparisons are needed. While undercollecting in the region limits our full understanding of the distribution of *B.occidentalis*, it is likely discontinuous due to altitude barriers and the patchy nature of SDTF. Its dispersal ability via ballistochory is limited, and its seeds even lack caruncles which are often implicated in Euphorbiaceae secondary seed dispersal by ants. Disjunctions across South America are known within and among closely related SDTF taxa, and in particular the legume species pair *Pithecellobiumdiversifolium* Benth. and *P.excelsum* (Kunth) Benth. resembles the transcontinental distribution of *Bahiana* (Lewis et al. 2006; Colli-Silva et al. 2021). Other legumes display stepping stone patterns either through southern SDTFs or northern connections. Patterns of Euphorbiaceae distribution and diversification across the SDTFs are largely unstudied beyond *Euphorbia*. A disjunct *Euphorbia* species pair – *E.heterodoxa* Müll.Arg. of eastern Brasil SDTF and *E.lagunillarum* Croizat of the dry Venezuelan Andes – with a distribution pattern approaching that of *Bahiana* suggests northern connections (Hurbath et al. 2021). *Gymnanthesboticario* and *Crotonlaceratoglandulosus* Caruzo & Cordeiro have disjunct distributions that follow the southern SDTF ecosystems from eastern Brazil to Bolivia (de Oliveira et al. 2013).

### ﻿Spinescence in Euphorbiaceae

Many Euphorbiaceae have well-developed intrinsic physical and/or chemical anti-herbivory defenses that include latex, toxic secondary chemistry, stinging trichomes, and spinescence. Escalation of plant defenses through multiple defense types rather than just refinements of a single type may be a recurring pattern in Euphorbiaceae (e.g., *Dalechampia*, Armbruster et al. 2009). Spinescence is generally associated with drier and more open habitats that can have abundant mammal herbivores (Charles-Dominique et al. 2016). Here, spinescence is considered broadly to include all sharp, hardened structures, thereby avoiding the inconsistent usage and not always clear distinctions among traditional definitions of spines (modified leaves or stipules), thorns (reduced branches), and prickles (emergences with epidermal-subepidermal origin and usually detachable) (see Bell 2008). My survey found vegetative spines in 25 genera of Euphorbiaceae scattered in subfamilies Acalyphoideae, Crotonoideae, and Euphorbioideae (Table 1). Their distribution is phylogenetically dispersed (relative to trees in Wurdack et al. [2005] and not explicitly mapped here) such that each genus has evidently evolved spines one or more times. Of the ca. 590 spiny species, 500+ are in *Euphorbia* L., the largest genus in the family, and the remaining genera mostly have a few spiny taxa each. The other two large genera of Euphorbiaceae, *Croton* and *Acalypha*, are notable for their paucity of spines despite considerable diversification in arid and open environments.

**Table 1.** Taxa of spinescent Euphorbiaceae. Taxonomy follows World Checklist of Selected Plant Families (WCSP 2018), except as noted, and the species of *Sebastiania* and *Gymnanthes* listed here are known to need generic adjustments.

Genus	# spinescent species/total species	Spinescent species	Distribution of spinescent species	Spine location or origins	Additional references
**Subfamily Acalyphoideae**					
*Acalypha*	3/ca. 500	*baretiae* I.Montero & Cardiel, *echinus* Pax & K.Hoffm., *sonderiana* Müll.Arg.	Madagascar, tropical East Africa	Leafy branch tips; possibly result of weathering	Muñoz et al. 2018
*Acidoton*	2/5	*microphyllus* Urb., *variifolius* Urb. & Ekman	Hispaniola	Lateral leafy branch tips	
*Adelia*	6/10	*barbinervis* Schltdl. & Cham., *brandegeei* V.W.Steinm., *membranifolia* (Müll.Arg.) Chodat & Hassl., *ricinella* L., *triloba* (Müll.Arg.) Hemsl., *vaseyi* (J.M.Coult.) Pax & K.Hoffm.	USA (Texas), Caribbean, Mexico, Central & South America	Branch tips	
*Alchornea*	1/51	*ilicifolia* (Js.Sm.) Müll.Arg.	Australia	Leaf margins	
*Bahiana*	2/2	*occidentalis* K.Wurdack, *pyriformis* J.F.Carrión	Brazil, Peru	Stipules	Carrión et al. 2022
*Bernardia*	1/80	*hamadryadica* J.F.Carrión & Cordeiro	Brazil	Lateral leafy branch tips	Carrión et al. 2017
*Caperonia*	3/34	*aculeolata* Müll.Arg., *buettneriacea* Müll.Arg., *heteropetala* Didr.	Brazil	Emergences as prickles along stems and abaxial midveins; well-developed in listed species but as small prickles or glandular trichomes in others	
*Doryxylon*	1/1	*spinosum* Zoll.	Malesia (Philippines, Lesser Sunda Islands)	Leafless axillary shoots	Welzen 1999
*Enriquebeltrania*	2/2	*crenatifolia* (Miranda) Rzed., *disjuncta* De-Nova & Sosa	Mexico	Lateral leafy branch tips; stipules are indurated, persistent but sub-spinose	
*Erythrococca*	ca. 10/41	Most members of sects. *Deflersia*, *Tristis*, *Lasiococcae* including: *E.anomala* (Juss. ex Poir.) Prain, *berberidea* Prain, *bongensis* Pax, *fischeri* Pax, *laurentii* Prain, *natalensis* Prain, *poggeophyton* Prain, *pubescens* Radcl.Sm., *subspicata* Prain, *zambesiaca* Prain	Africa	Stipules	Prain 1911; Uhlarz 1978
*Lasiocroton*	1/26	*microphyllus* (A.Rich.) Jestrow	Cuba	Lateral leafy branch tips	
*Macaranga*	18/ca. 260	*angolensis* (Müll.Arg.) Müll.Arg., *assas* Amougou, *barteri* Müll.Arg., *beillei* Prain, *capensis* (Baill.Sim, *heterophylla* (Müll.Arg.) Müll.Arg., *heudelotii* Baill., *klaineana* Pierre ex Prain, *longipetiolata* De Wild., *monandra* Müll.Arg., *occidentalis* (Müll.Arg.) Müll.Arg., *paxii* Prain, *pierreana* Prain, *poggei* Pax, *saccifera* Pax, *schweinfurthii* Pax, *spinosa* Müll.Arg., *staudtii* Pax	Africa (especially tropical west Africa)	Along trunk and branches as simple or divided structures	Whitmore 2008
*Philyra*	1/1	*brasiliensis* Klotzsch	Argentina, Brazil, Paraguay	Intrastipular structures; stipules are not spinose	
**Subfamily Crotonoideae**					
*Acidocroton* (including *Ophellantha*)	12/14	*acunae* Borhidi & O.Muñiz, *adelioides* Griseb., *ekmanii* Urb., *gentryi* Fern.Alonso & R.Jaram., *horridus* Urb. & Ekman, *litoralis* Urb. & Ekman, *lobulatus* Urb., *montanus* Urb. & Ekman, *oligostemon* Urb., *spinosus* (Standl.) G.L.Webster, *trichophyllus* Urb., *verrucosus* Urb.	Caribbean (especially Hispaniola), Central America	Stipules	Uhlarz 1978
*Croton*	2/1200+	*bispinosus* C.Wright, *brittonianus* Carabia	Cuba	Axillary leafless shoots. Spines on *C.bispinosus* can be 0–2 per axil	
*Jatropha*	ca. 10/174	Sects. Collenucia and Spinosa, including: *collina* Thulin, *dichtar* J.F.Macbr, *ellenbeckii* Pax, *glauca* Vahl, *humifusa* Thulin, *inaequispina* Thulin, *marginata* Chiov., *nogalensis* Chiov., *paradoxa* (Choiv.) Chiov., *rivae* Pax, *rosea* Radcl.-Sm., *tetracantha* Chiov.	East Africa (especially Somalia)	Stipules as simple or branched structures	Hemming and Radcliffe-Smith 1987; Thulin 1993
**Subfamily Euphorbioideae**					
*Bonania*	2/7	*domingensis* (Urb.) Urb., *elliptica* var. spinosa (Urb.) Borhidi	Cuba, Hispaniola	Lateral leafy branch tips	
*Euphorbia*	ca. 500+/2200	Parts of sects. *Goniostema*, *Monadenium*, *Euphorbia*, *Tirucalli* (*E.stenoclada* Baill.), *Espinosae* (*E.espinosa* Pax), *Articulofruticosae*	Old World xerophytes (especially Africa)	Stipules, leafy branch tips, tubercules (spine shields), and/or persistent peduncles	White et al. 1941; Uhlarz 1974; Yang et al. 2012; Dorsey et al. 2013; Peirson et al. 2013
*Gymnanthes*	3/25	*guyanensis* Müll.Arg., *hirsuta* Esser, *microphylla* Esser	Bolivia, Columbia, Guyana	Lateral leafy branch tips	
*Hippomane*	2/3	*horrida* Urb. & Ekman, *spinosa* L.	Hispaniola	Leaf margins	
*Hura*	2/2	*crepitans* L., *polyandra* Baill.	Mexico, Caribbean, Central & South America	Along trunk and branches, where they begin developing in young saplings and emerge by piercing the bark from subsurface (endogenous) origins	Lefebvre et al. 2022
*Pachystroma*	1/1	*longifolium* (Nees) I.M.Johnst.	Bolivia, Brazil, Peru, Paraguay	Leaf margins, variable 0–15 spines per side	
*Sebastiania*	8/56	*chaetodonta* Müll.Arg., *klotzschiana* (Müll.Arg.) Müll.Arg., *mosenii* Pax & K.Hoffm., *obtusifolia* Pax & K.Hoffm., *picardae* Urb., *schottiana* (Müll.Arg.) Müll.Arg., *serrata* (Baill. ex Müll.Arg.) Müll.Arg., *vestita* Müll.Arg.	South America, Caribbean (Hispaniola)	Branch tips. Spines well-developed in *S.picardae* and erratic in other species on leafy lateral branches	
*Spegazziniophytum*	1/1	*patagonicum* (Speg.) Esser	Argentina (Patagonia)	Branch tips (stems photosynthetic and leaves ephemeral)	
*Tetraplandra* (united with *Algernonia*, but the species here has yet to be transferred)	1/13	*anomala* Pax & K.Hoffm. (poorly known and possibly extinct species)	Brazil	Rarely present as leafless lateral branch tips (*Glaziou 8323*, K)	

**Figure 6.**
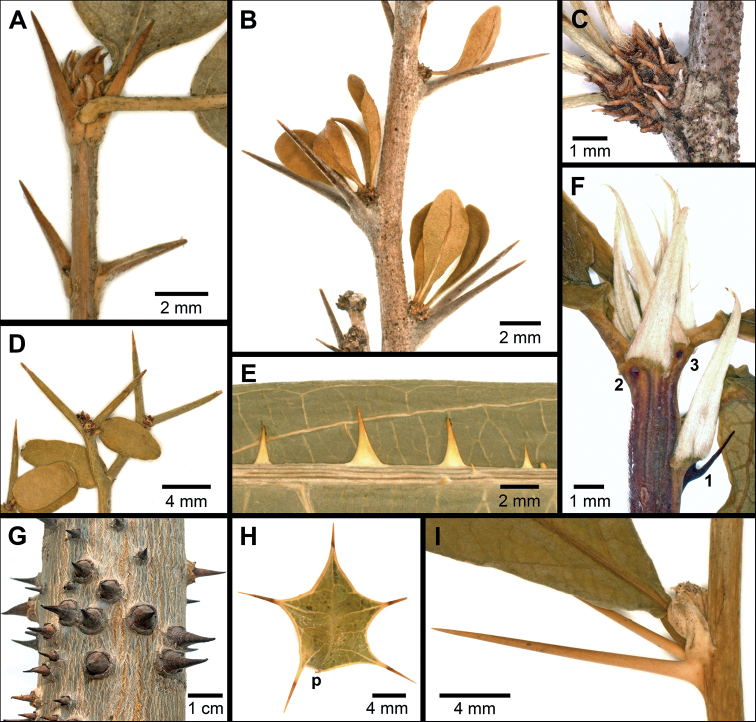
Diversity of spiny structures on Euphorbiaceae**A** spinose stipules and protected terminal resting bud (*Bahianaoccidentalis*, *C. Díaz S. et al. 5072*, MO) **B** spinose stipules subtending leafy fascicles (*Acidocrotonlitoralis*, *G. Proctor 10991*, US) **C** spinose stipules as clear pairs associated with each fascicle leaf (*Acidocrotonverrucosus*, *G. Webster et al. 8463*, US) **D** spinose branch tips (*Sebastianiapicardae*, *E. Ekman 2229*, US) **E** spiny emergences along primary vein (abaxial, *Caperoniabuettneriacea*, *G. Hatschbach 6394*, US) **F** intrastipular spine development (sequentially 1–3) in *Philyra* below stipule pairs at shoot tip (*P.brasiliensis*, *A. Gentry et al. 51884*, MO) **G** spiny emergences on trunk (*Huracrepitans*) **H** spiny leaf margins, p = site of petiole attachment below gland (*Hippomanehorrida*, *A. Liogier 14212*, US) **I** intrastipular spines mature and lignified in *Philyra* (same branch as **F**).

The spines in Euphorbiaceae have diverse origins (homologies) and positions on the plant, including modified branch tips (stem spines), stipules, peduncles, leaf margins, and stem emergences (Fig. 6). Leaf spines (i.e., an entire leaf taking the form of a spine) appear to be absent. The short leafless axillary spines in *Croton* L. (and *Doryxylon* Zoll.) could represent modified prophylls of axillary buds, but the replacement of spines with inflorescences on *Crotonbispinosus* C.Wright specimens (e.g., *C. Morton & J. Acuña 2964*, US) suggests they are fundamentally shoot axes. Most spiny taxa occur in dry and/or open environments (i.e., SDTF, scrub, and deserts), with the exceptions of *Hura* L. and *Macaranga* Thouars which are primarily in wet forests and *Caperonia* A.St.-Hil. in wetlands. When considering habit, nearly all spiny genera are woody shrubs or trees, with the exceptions being many *Euphorbia* spp. that are succulent (sometimes also woody), and *Caperonia* that are herbaceous. Stem spines, widely distributed across 12 genera, can be very plastic in their occurrence on individual plants, from terminating all lateral short shoots to an erratic distribution (sparse or sometimes with varying degrees of sharpness). The spines along the trunk and/or branches of *Hura* (Fig. 6G; Lefebvre et al. 2022) and *Macaranga* spp. (Jeník and Harris 1969; Whitmore 2008) need further study on their development; in the former they are considered cork-spines derived from suber and the latter derived from roots. Emergences, as sharp structures with epidermal-subepidermal origin (Bell 2008) on stems or leaves, are prominent in species of at least three genera. While toothed leaf margins occur widely across the family, strongly spinose teeth appear rare, although subspinose or mucronate intermediates exist (e.g., the teeth of *Alchorneacastaneifolia* [Humb. & Bonpl. ex Willd.] A.Juss. sometimes resemble less spinose margins of *A.ilicifolia* [J.Sm.] Müll.Arg.). Trichomes are not treated here; however, I note that they can be substantial and spinose in *Cnidoscolus* (e.g., *C.quercifolius* Pohl) (see Maya‐Lastra and Steinmann 2019). In some South American *Cnidoscolus* (e.g., *C.bahianus* [Ule] Pax & K.Hoffm., *C.pavonianus* [Müll.Arg.] Fern.Casas, *C.ulei* [Pax] Pax) bark forms around the trichome base as a turbinate collar and permanently anchors the usually detachable stinging arm. Spines of stipular origin, of special focus here and further detailed below, occur in five genera (*Acidocroton* Griseb., *Bahiana*, *Erythrococca* Benth., *Euphorbia*, *Jatropha* L.). *Acidocroton* and *Bahiana* are restricted to the New World, while the other three genera either are restricted to the Old World (*Erythrococca*) or have spiny species only there (*Euphorbia*, *Jatropha*).

*Acidocroton* (including *Ophellantha*) has paired spines of stipular origin that are usually 1–2× the length of the leaves (to 15 mm) in the microphyllous Caribbean species and much shorter (to 5 mm) in the large leaved taxa referred to sect. Ophellantha. A node subtended by a pair of long, strongly attached primary spines (i.e., those subtending the fascicle and that are usually larger) typically contains a cluster of fascicled leaves (up to 11 leaves per fascicle in *A.oligostemon* Urb.), long trichomes, and tiny spines which represent stipules for the other leaves (Fig. 6B). The spines precociously develop and well arm the branch tips (sometimes before the leaves expand) and then continue to enlarge and lignify. The spines in Jamaican *A.verrucosus* Urb. often show less dimorphism where the primary spines are often of similar short length (<1.5 mm) to those in the fascicle; they also show spine pairings with fascicle leaves that clearly indicate stipular origins (Fig. 6C).

*Bahiana* has paired spines of stipular origin that elongate, lignify, and often spread with age (Fig. 6A). Their prominence varies across collections in length and degree of spreading. Resting buds usually have nested sets of scales with spinose tips and overlapping papery basal margins. In *B.occidentalis* the spine core contains thin walled-cells and can be hollow towards the base (Fig. 5D).

The paired stipules of *Erythrococca* can be spinose and 1–5 mm long, but there is much variation among species as well-summarized by Prain (1911: 848) as “stipules cartilaginous, glabrous, often accrescent and modified into umbonate mammillae or weakly conical thorns, rarely into wide-based pungent spines, sometimes minute, subulate and unaltered”. In spinose *E.anomala* (Juss. ex Poir) Prain, they are asymmetric horns resembling rose thorns to 3 mm long, and they easily detach due to their basally hollow structure (see Uhlarz 1978).

*Euphorbia* spp. with their remarkable variation in xerophytic growth forms have equally diverse and complex spines, including stipules, modified branch tips, persistent peduncles, and tubercules (spine shields) bearing single, paired, or clustered spines of sometimes unclear origins (White et al. 1941; Uhlarz 1974). The spines can reach 7.5 cm long and be simple or elaborated with short branches. Uhlarz (1974) studied *Euphorbia* spine structure and ontogeny, and noted that “dorsal spines” (on the dorsal side of the leaf base) develop after the stipules and their formation is influenced by environmental factors such as light. There are an estimated 500+ spiny species, especially in subgen. Euphorbiasect.Euphorbia with more than 340 species, mostly with spine shields and spinose stipules. Spines (non-stipular) have evolved in subgen. Athymalus (sects. *Lyciopsis*, *Anthacanthae*) and subgen. Chamaesyce (sects. *Espinosae*, *Articulofruticosae*), but they are apparently absent in subgen. Esula (Yang et al. 2012; Dorsey et al. 2013; Peirson et al. 2013).

The stipules of *Jatropha* spp. are typically glandular (or reduced) but can be variously elaborated as spines in the northeast African species; they are not spinose in South African, Malagasy, or New World taxa. These stipular spines are stout and simple, up to 5 cm long (e.g., *J.dichtar* J.F.Macbr.), or thin and branched so as to cover the stems in spiny thickets (e.g., *J.marginata* Chiov.). Somalia contains an especially rich diversity of spiny-stipuled *Jatropha*, although some species distributed there are clearly not spinose (Hemming and Radcliffe-Smith 1987; Thulin 1993).

The paired intrastipular spines of *Philyrabrasiliensis* Klotz. are outgrowths on each side of the petiole base just below (and distinctly separate when young) the persistent stipules. The spines usually develop (sometimes starting as a pigmented spot) near the shoot tips after the young leaves have begun to expand, and then lignify and elongate up to 3 cm (Fig. 6F, I). There is considerable plasticity in presence and mature spine length across branches and collections, and sometimes the spines are scarcely evident. While not stipular in origin, their homologies are unclear. They appear to be similar to intrastipular spines described in some legumes and suggested to be either modified short shoots (Sharma and Kumar 2012; Juárez et al. 2018) or emergences (Bell 2008).

## ﻿Conclusions

*Bahianaoccidentalis* is a distinct new species that broadens the character states for the genus, notably in inflorescence structure details and in fruit more typical of Euphorbiaceae in size and shape. The distribution of *Bahiana* is unusual and adds to emerging patterns of SDTF flora disjunctions, although its transcontinental nature is not informative as to whether this arose from northern or southern dispersal routes. While legumes are the most species-rich and investigated component of SDTF floras, Euphorbiaceae deserve further study, and *Bahiana* demonstrates that surprises remain. Spines in Euphorbiaceae are diverse in origin, and with relatively few exceptions occur in woody or succulent taxa from dry or open environments. While the focus here is on spines from a family perspective, their occurrence in the SDTF flora deserves further study and quantification (legumes and cacti are notable spiny components).

## Supplementary Material

XML Treatment for Bahiana occidentalis
